# Clinical efficacy of conbercept plus micropulse laser (577 nm) treatment in macular edema secondary to non-ischemic central retinal vein occlusion

**DOI:** 10.12669/pjms.38.5.5231

**Published:** 2022

**Authors:** Li Li, Qian Ren, Zhaohui Sun, Hua Yu

**Affiliations:** 1 Li Li, Department of Ophthalmology, Shijiazhuang People’s Hospital, Shijiazhuang, 050011, Hebei, China; 2 Qian Ren, Department of Ophthalmology, Shijiazhuang People’s Hospital, Shijiazhuang, 050011, Hebei, China; 3 Zhaohui Sun, Department of Ophthalmology, Shijiazhuang People’s Hospital, Shijiazhuang, 050011, Hebei, China; 4 Hua Yu, Department of Ophthalmology, Shijiazhuang People’s Hospital, Shijiazhuang, 050011, Hebei, China

**Keywords:** Central retinal vein occlusion, Macular edema, Conbercept, Micropulse laser

## Abstract

**Objectives:**

To observe the clinical efficacy and complications of conbercept combined with a 577-nm micropulse laser in patients with macular edema (ME) secondary to non-ischemic central retinal vein occlusion (CRVO).

**Methods:**

A total of 153 patients who were diagnosed with non-ischemic CRVO-induced ME and treated by Shijiazhuang People’s Hospital during January 2019 and January 2021 were included in this study. The patients were divided into a control group and an experimental group by choice of treatment. The control group was treated by conbercept alone, while the experimental group underwent laser treatment on this basis. The best corrected visual acuity (BCVA) was determined by the internationally standardized logarithm of the minimum angle of resolution (logMAR) chart and the central macular thickness (CMT) was measured by optical coherence tomography (OCT) before and at one and three months after treatment. Complications such as conjunctival hemorrhage and elevated intraocular pressure (if any) were recorded during the 3-month follow-up period.

**Results:**

Conbercept could improve the BCVA and CMT of patients with non-ischemic CRVO (P <0.05, respectively), and the combined use of conbercept with micropulse laser treatment yielded greater improvements in these measures compared with the pre-treatment condition (P <0.05, respectively); moreover, the differences between the control group and the experimental group were statistically significant (all P <0.05). The two groups had similar complication rates and did not record any serious adverse reactions.

**Conclusions:**

Conbercept combined with 577-nm micropulse laser treatment can benefit patients with non-ischemic CRVO-induced ME by improving their visual acuity and relieve ME symptoms. As a regimen of impressive clinical efficacy, it is of great value for wide application in clinical practice.

## INTRODUCTION

Central retinal vein occlusion (CRVO) is a retinal vascular disorder, and non-ischemic CRVO is a clinically common condition that can lead to macular edema (ME), a major cause of visual impairment and even loss of visual acuity.^1^-^3^ ME patients were largely administered with conbercept, a vascular endothelial growth factor (VEGF) inhibitor proven to produce desired therapeutic effect in treating neovascularization in fundus and relieve ME based on a multiple-dosing regimen.^4^-^6^

Micropulse laser treatment is a novel minimally invasive approach that helps improve retinal pigment epithelial function and reduce ME by acting on the retinal pigment epithelium, without causing any pain, severe injury or damage to visual function.^7^-^9^ In this study, a regimen combining conbercept with a 577-nm micropulse laser to treat non-ischemic CRVO-induced ME was introduced to observe its efficacy and complications.

## METHODS

This study included 153 patients with non-ischemic CRVO-induced ME from Shijiazhuang People’s Hospital during January 2019 and January 2021 who were assigned to a control group and an experimental group according to their treatment strategies. The sample size required for each group was calculated by the formula 
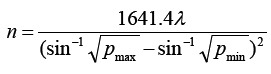
. The control group was treated with conbercept alone, while the experimental group was given conbercept in combination with laser treatment. This study has been approved by the Ethics Committee of Shijiazhuang People’s Hospital. The control group (n =78) was comprised of 43 males and 35 females, with the mean age of (46.23 ±9.59) years (range: 31-58 years) and the mean course of disease of (6.01 ±3.16) months (range: 1-11 months). The experimental group (n =75) consisted of 38 males and 37 females, with the mean age of (44.45 ±10.67) years (range: 29-60 years) and the mean course of disease of (5.94 ±3.02) months (range: 1-11 months). General information did not differ significantly between the two groups (P >0.05).

### Ethical Approval:

The study was approved by the Institutional Ethics Committee of Shijiazhuang People’s Hospital on May 15, 2019 (No.[2019]051), and written informed consent was obtained from all participants.

### Inclusion Criteria:

- A patient was rendered eligible for this study if he/she
- Was confirmed to have non-ischemic CRVO accompanied with ME based on hemorheological analysis, fluorescein angiography of the ocular fundus and optical coherence tomography (OCT);
- Was not allergic to conbercept;
- Had clear dioptric media and was suitable for laser treatment;
- Showed good patient compliance and had no surgical contraindication.

### Exclusion Criteria:

- A patient was excluded if he/she
- Was pregnant or breastfeeding;
- Had recently received vitrectomy;
- Had diabetic optic neuropathy; 4) had mental disorders or any other diseases suggesting intolerance of surgical treatment.

### Control Group:

Each patient in the control group was administered with conbercept (Chengdu Kanghong Biotechnology Co., Ltd., G.Y.ZH. Zi S20130012) solely. Following standard preparation and anesthesia, the patient was placed in the supine position. Sterilization and draping was performed in the conventional manner. The eyelid was kept open using an eye speculum to wash the conjunctival sac with 0.9% injectable sodium chloride. Conbercept (0.05 mL) was carefully injected into the vitreous cavity in a vertical direction at the sclera 3.5 mm from the conjunctiva. After that, the site of injection was pressed with a wet cotton swab for seconds, followed by pressure dressing with sterile gauze, which was removed the next day. Then, the patient was prescribed with levofloxacin eye drops, four times daily for three days.

### Experimental Group:

In addition to injection with conbercept, the experimental group received 577-nm micropulse laser treatment using a laser system (Iridex Corp., USA) with the micropulse laser mode, in which the spot size was between 100-200 μm and the exposure duration was 0.2 s. Procedures: Semicircular photocoagulation was performed upward or downward at 500-750 μm from the central foveola of the macula. Quadrant photocoagulation with conventional 557-nm continuous-wave lasers was applied to the affected area. For smaller lesions, quadrant photocoagulation and macular micropulse laser treatment were conducted simultaneously; for larger lesions, quadrant photocoagulation was initiated following macular micropulse laser treatment.

### Outcome Measures:

The best corrected visual acuity (BCVA) was determined by the internationally standardized logarithm of the minimum angle of resolution (logMAR) chart and the central macular thickness (CMT) was measured by optical coherence tomography (OCT) before and at one and three months after treatment. Complications such as conjunctival hemorrhage and elevated intraocular pressure (if any) were recorded during the 3-month follow-up period.

### Statistical Analysis:

The software SPSS19.0 was used for statistical analysis. Enumeration data were expressed by percentage (%) and intergroup comparison was examined by the contingency table chi-square test. Measurement data were represented by (x±s) and analyzed by the paired sample t-test. Statistical significance was set at P <0.05.

## RESULTS

The two groups showed no significant difference in BCVA (P >0.05) and experienced marked improvement in BCVA one and three months after treatment (P <0.05, respectively). Moreover, greater improvement was observed in the experimental group when compared with the control group (P <0.05) (Table-I).

**Table I:** Comparison of visual acuity improvement between the two groups.

Group	Number of Patients	Before Treatment	1 Month after Treatment	3 Month after Treatment
Control group	78	0.29±0.04	0.38±0.02*	0.42±0.03*
Experimental group	75	0.3±0.03	0.42±0.03*#	0.46±0.02*#

* P <0.05 compared with the pre-treatment condition; #P <0.05 compared with the control group.

Before treatment, the two groups showed no significant difference in CMT (P >0.05). After one and three months of treatment with conbercept or conbercept plus micropulse lasers, CMT was reduced in both groups compared with the pre-treatment level (P <0.05, respectively), and the differences between the two groups were statistically significant (P <0.05, respectively) (Table-II).

**Table II:** Comparison of CMT improvement between the two groups.

Group	Number of Patients	Before Treatment	1 Month after Treatment	3 Month after Treatment
Control group	78	559.45±9.24	383.65±14.37*	272.18±14.02*
Experimental group	75	560.96±11.14	247.51±13.22*#	221.56±10.84*#

* P <0.05 compared with the pre-treatment condition; #P <0.05 compared with the control group.

Complications that occurred during the course of treatment mainly included conjunctival hemorrhage and elevated intraocular pressure; complication rates were low in both groups, and the difference between the two groups lacked statistical significance (P >0.05) (Table-III).

**Table III:** Comparison of complication rate (%) between the two groups.

Group	Number of Patients	Conjunctival Hemorrhage	Elevated Intraocular Pressure	Complication Rate
Control group	78	2 (2.56)	3 (3.84)	5 (6.41)
Experimental group	75	2 (2.67)	2 (2.67)	4 (5.33)

## DISCUSSION

CRVO is a clinically common vessel occlusion of the ocular fundus that can be caused by numerous factors. The disease usually affects one eye only and mostly occurs in patients with glaucoma. CRVO is generally manifested by diffuse flame-shaped and splinter hemorrhage in the retina at the ocular fundus, and sometimes occurs in parallel with cystoid ME or secondary neovascular glaucoma.^1^-^3^ CRVO patients are at high risk of retinal hemorrhage and ME, which are leading causes of decreased vision.^1^-^3^,^10^ As a serious complication of non-ischemic CRVO, ME can induce cell apoptosis and retinal fibrosis in the long run if the patient cannot receive appropriate treatment in a timely manner, which can lead to irreversible visual impairment and adversely affect the quality of life.^10^,^11^ The pathogenesis of CRVO-induced ME is still unclear and yet, it is generally believed to be closely associated with damage to the blood-retinal barrier. CRVO brings structural and functional changes to the retinal capillaries and damages the blood-retinal barrier by inducing varying degrees of hypoxia in the retina, eventually leading to retinal edema as macromolecules pass through the capillaries into the retina.^1^-^3^,^10^,^11^ Hypoxia and ischemia in the retina can increase VEGF expression, promote vasopermeability and result in ME, which entails a risk of loss of visual acuity.^11^-^13^

CRVO is treated in the principle of controlling bleeding and ME as well as prevention and control of complications through intravitreal injection of drugs, laser treatment and surgery.^1^-^3^ Conbercept, a self-developed anti-VEGF agent in China, is essentially a recombinant fusion protein that inhibits neovascularization by acting on VEGFs through specific binding to prevent them from binding to their receptors (VEGFRs). Conbercept not only enables extensive inhibition of neovascularization and inflammatory response but also reduces damage to retinal capillary endothelial cells, effusion and retinal edema, thereby inhibiting capillary leak, resolving retinal and macular edema and improving visual acuity.^4^-^6^ Results of our study also showed that conbercept could effectively improve the BCVA and CMT of patients with non-ischemic CRVO, which was similar to the results of previous studies. Studies have demonstrated that intravitreal injection of conbercept has satisfactory short-term efficacy and safety in treating secondary ME; however, the recurrence rate is relatively high and multiple injections are required, which brings a heavy financial burden and physical pain to patients.^14^,^15^

The 577-nm wavelength yellow micropulse laser is widely used in clinical practice because of its advantages such as low light scattering, negligible xanthophyll absorption and excellent combined absorption by melanin and oxyhemoglobin. Moreover, this high-frequency continuous-wave laser can accurately act on the retinal pigment epithelium without affecting the choroid membrane or neuroepithelium. In addition, it is effective in preventing formation of scars and neovascularization. Therefore, this technique is ideal for patients with non-ischemic CRVO-induced ME and is shown to yield better outcomes when used in combination with anti-VEGF agents.^9^,^16^ In a prospective study of patients with diabetic macular edema who did not respond to anti-VEGF therapy, micropulsed laser therapy improved BCVA and CMT in patients with anti-VEGF-resistant diabetes.^17^ The results of Bougatsou P et al.^18^ also showed that micropulse laser has a significant role in the treatment of non-centrally involved and clinically significant macular edema. Our results also found that conbercept combined with micropulsed laser therapy was more effective in improving BCVA and CMT in patients with non-ischemic CRVO, which was similar to the results of previous studies. In another prospective study,^19^ 23 eyes (33 eyes) with treatment-naïve or refractory DME were treated with high-density micropulses using the Easy Ret 577 semiconductor laser for three months, and no significant improvement in BCVA or macular thickness was observed. This was not consistent with the conclusion of our study. This difference may be related to the small sample size included in their study or the refractory cases of their study subjects. Notably, in our study, complication rates were similar in both settings, and no serious adverse reaction was observed.

### Limitations of the study:

The number of subjects included in this study was limited, so the conclusions drawn may not be very convincing. In addition, this study was a retrospective study with limited data integrity and homogeneity. It is necessary to further design a randomized controlled trial to verify the conclusions of this study.

## CONCLUSION

Conbercept plus 577-nm micropulse laser treatment as a combined regimen for patients with non-ischemic CRVO-induced ME can markedly relieve ME symptoms and improve visual acuity. Considering its clinical efficacy, the regimen is of enormous value in clinical practice.

### Authors’ Contributions:

**HY &** **LL:** Designed this study, prepared this manuscript, are responsible and accountable for the accuracy and integrity of the work.

**QR:** Collected, analyzed clinical data.

**ZS:** Significantly revised this manuscript.
